# Strengths of fertilizer and litter effects on seedling recruitment and growth of grassland species differ depending on functional groups and seed size

**DOI:** 10.1002/ece3.11650

**Published:** 2024-07-03

**Authors:** Sarah Martin, Christiane Roscher

**Affiliations:** ^1^ Department of Physiological Diversity UFZ, Helmholtz Centre for Environmental Research Leipzig Germany; ^2^ German Centre for Integrative Biodiversity Research (iDiv) Halle‐Jena‐Leipzig Leipzig Germany

**Keywords:** early life stage, fertilization, functional traits, germination, grassland, litter cover, nitrogen, seedlings

## Abstract

Agricultural grasslands play an important role in conserving the biodiversity of the European cultural landscape. Both, litter cover and soil nutrient availability, change with grassland management, but it is not well‐studied how seedling recruitment and growth of multiple grassland species are influenced by their single or combined effects. Therefore, we studied the effects of nitrogen fertilization (100 kg N per year and ha) and litter cover (250 g_dw_ per m^2^) on seedling recruitment and growth of 75 temperate grassland species (16 graminoid species, 51 forb species, 8 legume species) in a full factorial microcosm experiment. Overall, fertilizer reduced seedling emergence, while litter cover increased it even when combined with fertilization. Fertilization increased seedling height and biomass, and the combination of fertilizer and litter resulted in even stronger responses. Litter cover alone did not influence seedling biomass or seedling height. While the overall direction of treatment effects was similar across functional groups, their strengths were mostly weaker in graminoids than in non‐legume forbs and legumes. Positive litter effects on seedling emergence were stronger in large‐seeded species. Positive fertilization effects on seedling growth were stronger in small‐seeded species, while their seedling biomass was negatively affected by litter cover. In summary, our results show for multiple grassland species that the combination of litter cover and fertilization modulates their single effects. The varying sensitivity of how grassland species representing different functional groups and seed sizes respond with their seedling emergence and growth to litter cover and nitrogen fertilization indicates that the consequences of land‐use change on grassland diversity and composition already start to manifest in the earliest stages of the plant life cycle.

## Graphical abstract

Land use intensity has increased over the last decades influencing plant species composition. The regeneration niche has often been overlooked in this regard. However, seedling reaction in growth and biomass to litter and fertilizer addition differs depending on functional groups and seed size. This implies that land use already affects the earliest stages of the plant life cycle.

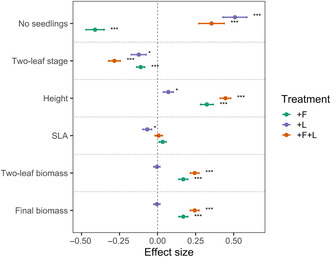

## 1 INTRODUCTION

Grasslands harbor large biodiversity and characterize Central Europe's cultural landscape covering approx. 20% of the land surface (Eurostat, 2018). Hence, grasslands are of major importance in nature conservation (Wesche et al., 2012) and deliver multiple ecosystem services (Bengtsson et al., 2019). However, several factors threaten grassland biodiversity, and land‐use change is one of the most important threats (Newbold et al., 2015; Sala et al., 2000). During the last century, the management intensity of many grasslands has been increased through fertilization, higher mowing frequency, or livestock density. Nitrogen fertilization is known to increase aboveground plant biomass production, reduce species richness, and alter species composition towards a higher proportion of grasses and fewer forb species (Čop et al., 2009; de Schrijver et al., 2011; Stevens et al., 2004). Species richness is not only threatened by high land‐use intensity but also by abandonment (Pruchniewicz, 2017). Low management intensity or abandonment promotes tall and productive species due to the accumulation of nutrients and increased competition for light and it is associated with the accumulation of dead plant material (Pavlů et al., 2012).

Plant regeneration is a key demographic process influencing species diversity and composition of plant communities. The regeneration niche of a plant species is defined by the conditions needed for germination and seedling growth (Grubb, 1977), which might diverge from the environmental conditions required for the growth of adult plant individuals (Poorter, 2007). The availability of microsites with suitable conditions is crucial for successful germination and seedling establishment (Aicher et al., 2011). In grasslands, management intensity might influence the availability of suitable microsites for seedling emergence and growth in different ways.

Nutrient availability, especially nitrogen, might affect germination differently (Fenner, 2000; Pérez‐Fernández et al., 2006). At low to moderate levels, nitrogen may promote germination either as a nutrient or as a signaling molecule (Duermeyer et al., 2018; Osuna, Prieto, & Aguilar, 2015). However, germination might be suppressed at higher nitrogen levels, possibly due to toxic effects, specific inhibitions, its interaction with phytohormones which regulate seed dormancy, or osmotic effects (Fenner, 2000; Osuna et al., 2015). However, these processes are very species dependent and so far, the molecular mechanisms have been mainly studied in model species (Duermeyer et al., 2018). Several studies indicated that the negative effects of nitrogen fertilization on species diversity are dependent on functional groups or species life histories resulting in a stronger loss of perennial non‐legume forb species, annuals, and biennials and legumes (Varma et al., 2016), while grass species are less affected (Foster & Gross, 1998; Zhong et al., 2019). Most of these studies have been conducted with few species, while it is not well studied how nitrogen fertilization affects seedling emergence and growth of a larger range of grassland species assigned to different functional groups.

More studies exist investigating the effects of litter cover on seedling emergence and growth (see meta‐analyses in Loydi et al., 2013; Zhang et al., 2022). In a meta‐analysis, Loydi et al. (2013) showed that low and medium amounts of litter exert positive effects on seedling recruitment and growth, while the effects of litter become negative above a certain amount of litter. Litter cover may act as a mechanical barrier to seedling emergence, reduce the quantity and affect the quality of light (Kahmen & Poschlod, 2008; Zhang et al., 2019), buffer soil moisture by reducing evaporation (Zhang et al., 2022), and influence soil temperature. On the one hand, litter can decrease temperature fluctuations at the soil surface and therefore, inhibit germination (Fenner, 2000). On the other hand, litter can also have facilitative effects on germination, for example by dampening temperature extremes and thereby preventing frost and heat damage (Hou et al., 2020). Chemically, litter decomposition may be a source of nutrients and other leachates which might be toxic or immobilize nutrients, but their effects on germination might vary among species or functional groups (Amatangelo et al., 2008; de Jong & Klinkhamer, 1985; Hovstad & Ohlson, 2008).

Apart from differences among plant functional groups, which might respond differently in their germination and seedling growth to nitrogen fertilization and litter cover, species responses might depend on seed size. Seed size is a key trait for the regeneration of grassland species (Moles & Westoby, 2004). It has been found to be a good predictor of the effects of litter on seedling emergence, but not for seedling biomass (Loydi et al., 2013). While seedling emergence was shown to be less affected by litter in large‐seeded species, small‐seeded species showed a negative response. Species with small‐sized seeds usually need light for germination (Baskin & Baskin, 2001; Milberg et al., 2000) and contain fewer nutrient reserves (Westoby et al., 1996), which might explain their reduced emergence under a litter cover. Small nutrient reserves in small‐sized seeds imply that fertilizer effects could vary among species with different seed sizes. Indeed, a previous study indicated that a large seed size is beneficial under nutrient‐poor conditions, while seedling growth under fertilized conditions did not vary depending on seed size (Bergholz et al., 2015).

Although the effects of litter on seedling emergence and growth are well studied (Loydi et al., 2013; Zhang et al., 2022), less is known about how nitrogen fertilization alone or in combination with litter affects seedling recruitment and growth of grassland species. Negative effects of litter accumulation on germination and seedling growth could be especially strong under nutrient‐rich conditions at medium to low management intensities when more litter accumulates due to greater productivity. At the same time, high soil nitrogen availability could also directly affect germination and seedling growth and, therefore, fertilization might have long‐term consequences for grassland restoration during extensification, especially when management intensity is reduced in formerly highly fertilized grasslands (Willems & van Nieuwstadt, 1996). Moreover, studies of seedling emergence of grassland species often focused on single or few species (e.g. Boudell & Stromberg, 2015; Ruprecht et al., 2010; Wellstein, 2012; Zhang et al., 2020). Negative effects of increased land‐use intensity, however, are not only restricted to a few particular species but lead to a general decline in grassland plant diversity (Busch et al., 2019; Gilhaus et al., 2017; Gossner et al., 2016; Socher et al., 2012). Therefore, it is a pressing question to understand how a broad range of common grassland species are affected in their earliest life stage and if functional groups and/or seed size can be used to predict their responses to litter and fertilization. In the present study, we conducted an experiment with 75 grassland species representing three commonly differentiated functional groups (16 graminoid species, 8 legume species, 51 non‐legume forb species) varying in their seed mass (from 0.04 to 26.97 mg per seed) to test the influence of litter and fertilizer and their combined effects on seedling recruitment and growth. We hypothesized that: (1) Both, nitrogen fertilization and litter cover alone affect seedling emergence and growth, however, their combined effects cannot be easily predicted from their single effects, but they depend on the interaction between both factors. (2) The single and combined effects of fertilization and litter on seedling emergence and growth of grassland species assigned to different functional groups vary in strength, but to a lesser degree in direction. (3) In addition to differences among functional groups, the effects of fertilization and litter on seedling emergence and growth vary depending on seed size.

## 2 MATERIALS AND METHODS

### 2.1 Study species

In total, 75 grassland species were used for the experiment (see Table S1 for a species list), which represented three functional groups (16 graminoid species, 51 non‐legume forb species, and 8 legume species) and which were used in the seed addition treatments of the land‐use reduction experiment of the Biodiversity Exploratories (Andraczek, Weigelt, Cantuarias, et al., 2023; Fischer et al., 2010). Seeds were obtained from commercial suppliers (Rieger‐Hofmann GmbH, Blaufelden‐Raboldshausen, Germany, and Wildsamen‐Insel, Temmen‐Ringenwalde, Germany) and represented regional provenances for the three regions of the Biodiversity Exploratories (North‐eastern Germany, Central Germany, and South‐western Germany). Seeds were stored in a freezer (at −20°C) until they were prepared for the experiment.

### 2.2 Experimental design

The microcosm experiment was carried out at the field station of the UFZ in Bad Lauchstädt (51.3923°, 11.8759°, 117 m a.s.l., Germany). The experiment had a full factorial design with fertilizer addition (two factor levels: no fertilization, and fertilization) and litter addition (two factor levels: no litter addition, and litter addition). This resulted in four treatments (control = C, fertilization = +F, litter = +L, fertilization, and litter = +F + L) for each of the 75 species which were replicated four times resulting in a total of 1200 microcosms. Pots (11 × 11 cm, 12 cm depth) were filled with commercial potting substrate (Fruhstorfer Erde®, Type P70, Industrie‐Erdenwerke Archut GmbH, Lauterbach, Germany) mixed with 25 vol% mineral sand. The mixture contained 38 mg kg^−1^ plant‐available phosphorus, and 256 mg kg^−1^ plant‐available potassium (both determined with the CAL method). Soil carbon concentration was 62.3 mg g^−1^, and total soil nitrogen concentration was 1.3 mg g^−1^. The pH (measured in a CaCl_2_ solution) was 5.9.

The experiment was sown between 1st and 8th November 2021. Sowing in late autumn guaranteed cold stratification and/or short days, which are necessary for several species to induce germination (Baskin & Baskin, 2001; Fenner, 2000). Each pot was sown with 60 seeds of a single species. When provenances of different regions were available, seed origins were mixed in equal proportions. Seeds were gently pressed on the soil surface and covered with a thin layer of mineral sand to avoid loss through wind. For the fertilization treatment, the fertilizer was added in two portions, the first directly at sowing, and the second at the start of the growing season (1st March 2022). Either 20 m of water or fertilizer solution (3.024 mg N per mL) was added with a dispenser, resulting in a total nitrogen addition of 100 kg N ha^−1^ per year which is a common amount of fertilizer application in agricultural grasslands (Blüthgen et al., 2012). Nitrogen was added as calammonium nitrate (76% NH_4_NO_3_, 24% CaCO_3_) (Triferto Fertilizers, Kalkammonsalpeter N27) which is typically used as mineral fertilizer in agriculture. The fertilizer was applied before the litter was added at the first application. For the second application, the litter cover was lifted to add the fertilizer underneath. For the litter treatment, 3 g_dw_ litter was added, approx. 3 cm high in each pot, which resembles 248 g_dw_ m^−2^, which is in the range typically found in extensively used grasslands (Donath et al., 2004). The grass litter was collected in non‐fertilized grasslands in the surroundings of the experimental field station in late summer 2021 and air‐dried. It mainly consisted of common grass species such as *Poa pratensis* L., *Lolium perenne* L., and *Elymus repens* L., which belong to the most abundant grass species in all regions of the Biodiversity Exploratories (Andraczek, Weigelt, Cantuarias, et al., 2023). The grass litter was manually chopped to pieces of approx. 3 cm in length. The C and N concentration of the litter was 453.4 mg g_dw_^−1^ and 10.6 mg g_dw_^−1^, respectively. Pots were partially buried in the ground to a depth of approx. 7 cm in beds filled with bark mulch organized in four experimental blocks, each containing one replicate of each species × treatment combination. Beds were covered with pond nets (15 mm mesh size) to prevent loss of litter through wind or damage by birds. Pots received water through precipitation and were not additionally watered during winter. On 28th February 2022, pots were brought into an open greenhouse with ambient temperatures and a glass roof that closes during rain. The pots were placed on greenhouse tables arranged in four experimental blocks as before. In the greenhouse, all pots were watered with tap water two times per week or every second day when evaporation increased in mid‐June.

To assess additional input of nitrogen through deposition or leaching from litter, ion exchange resins were used, placing resin bags on the soil surface either in pots without litter or with litter (each with four replicates). Pots were distributed in the experimental blocks (for details see Appendix S1). Contrary to our expectations, the amount of mineral nitrogen was lower with a litter cover than without (Figure S1). Relative air humidity (Rh), air temperature near the soil surface, and soil temperature at 4 cm depth were monitored by attaching loggers (HOBO Pro v2 Temp/RH, HOBO Pro v2 Temp/6′Ext Temp, onset®) to additional pots (each with four replicates – in case of temperature four with litter and four without litter, respectively) placed randomly in each experimental block. Soil temperature (daily means and variability) did not differ depending on litter cover (see Figure S2a–c).

### 2.3 Data collection

Starting on the 1st of March 2022, all pots were controlled monthly until May 2022 to count the number of seedlings (=no. seedlings). A seedling was counted as emerged when it had developed cotyledons or later stages with true leaves. Exceptions were *Lathyrus pratensis* L. and *Vicia cracca* L., which have cryptocotylar hypogeal cotyledons. The number of seedlings for these species was counted as soon as a shoot axis started to emerge from the seed.

When seedlings reached the stage of two fully expanded true leaves (or two leaf pairs in the case of species with opposite leaves) (=time two‐leaf stage), two experimental blocks were used to harvest four to five seedlings of each species × treatment combination. Seedlings were cut at ground level and transported in moistened tissue paper into the laboratory. There, the stretched height of each seedling was measured with a ruler (=height). After that, the leaves were scanned with a flatbed scanner (Epson Perfection V550 Photo, EPSON) at 300 dpi. Images were analyzed with the software Image J (Schneider et al., 2012) to obtain the leaf area. The scanned leaves and the remaining parts (stem, cotyledons) of the seedlings were separately dried at 70°C for 48 h and then weighed to obtain seedling biomass in the two‐leaf stage (=two‐leaf biomass). The leaf area divided by the dry mass of the scanned leaves resulted in the specific leaf area (mm^2^ mg_dw_^−1^; =SLA). Because the temporal development of the seedlings varied among treatments, the remaining two experimental blocks were used for a second harvest (=final biomass) cutting again five seedlings at ground level of each species × treatment combination 1 week after all treatment combinations of a certain species had been harvested for the two‐leaf biomass. Thus, our first biomass harvest represents a certain developmental stage of the seedlings, while the final biomass accounts for treatment‐dependent differences in seedling biomass at a certain point in time. These samples were also dried at 70°C for 48 h and then weighed. The experiment was terminated on 6th July 2022 when all species reached the “final biomass.” Two species (*Heracleum sphondylium* L., *Silaum silaus* (L.) Schinz et Thell.) failed to germinate completely, while only one or two individuals of two further species (*Sanguisorba officinalis* L., *Falcaria vulgaris* Bernh.) germinated. Consequently, these species were excluded from the analysis of the seedling counts, but the measured seedlings were kept in other analyses.

The seed mass of each species was determined by weighing three batches of 50 seeds, divided by the number of counted seeds to get the average seed mass (see Table S1 and Figure S6). When seeds of different regional provenances were available, seed mass was separately determined for each provenance and averaged (=seed mass).

### 2.4 Data analysis

Statistical analyses were carried out in R version 4.2.2 (R Core Team, 2008) using the package lme4 (Bates et al., 2015). The R code is publically available at bexis.uni‐jena.de (DOI: 10.25829/bexis.31687‐10). Linear mixed‐effects models were used to test for the effects of the experimental treatments (fertilizer, litter), and for differences depending on functional group identity. Our fixed effects were the experimental factors fertilization, litter, the interaction between fertilization and litter, functional group (graminoids, legumes, non‐legume forbs), and its interaction with the experimental factors. Random effects were pot (in case of variables measured at individual seedlings) nested in block or only block to account for the design of the experiment. Furthermore, we added an independent random effect for species identity. To test if species differ in their response to litter cover and fertilization, we followed the recommendation by Zuur et al. (2009) and started with a model with the full set of fixed effects and first decided if our random effects should include only species identity, or should be extended by incorporating the interaction of species identity with fertilization or litter alone, or both. Models were fitted with restricted maximum likelihood because we were most interested in the random effects (Zuur et al., 2009). Model selection was based on Akaike's information criterion (AIC) (Burnham & Anderson, 2004) and showed for all variables except SLA and days to two‐leaf stage that the models including an interaction of species identity with fertilization and with litter represented our data best. Thus, we kept the best structure of random effects for each model individually and added the fixed effects sequentially as described above. Models were fitted with maximum likelihood and likelihood ratio tests were used to assess the statistical significance of the fixed effects. All continuous variables were log‐transformed to reach a normal distribution of within‐group errors and homogeneity of variances. The treatment effects on the number of emerged seedlings were tested with a generalized mixed‐effects model with a Poisson distribution. Differences among functional groups or their interactions with the experimental factors were identified with a post hoc test calculating the pairwise contrasts of the estimated marginal mean with the *emmeans* package (Lenth, 2023). To test for possible effects of seedling density on the measured growth‐related variables, the number of seedlings growing in a pot (square‐root transformed) was entered as a covariate before the experimental factors and compared with the models without seedling densities. Seedling density was significant in the case of the number of days until the two‐leaf stage was reached, biomass at the two‐leaf stage and SLA, with the respective random structure. However, the inclusion of seedling density did not change the results concerning the experimental factors in the case of the two‐leaf biomass nor the number of days to reach the two‐leaf stage, while for SLA litter was not significant anymore when accounting for seedling density (see Table S2).

Species intercepts and treatment response slopes for fertilization and litter (from the random part of the full models) were regressed against seed mass (log‐transformed) to test if seed mass could explain species‐level differences in the measured variables and varying response strengths to the fertilizer and litter treatment.

To compare treatment effects on the different measured variables, effect sizes were calculated as log response ratios (Hedges et al., 1999). First, data were corrected for possible effects of the experimental blocks. Because not all values remained positive after the correction for block effects, a small number was added to each value of a variable making the lowest number of the variable positive. Afterward, effect sizes (ln RR) were calculated for each species by dividing the species mean in a particular treatment (i.e. fertilizer addition, litter addition, or both) by the species mean of the control (i.e. without fertilizer and litter addition).

## 3 RESULTS

### 3.1 Seedling number and growth

**FIGURE 1:**
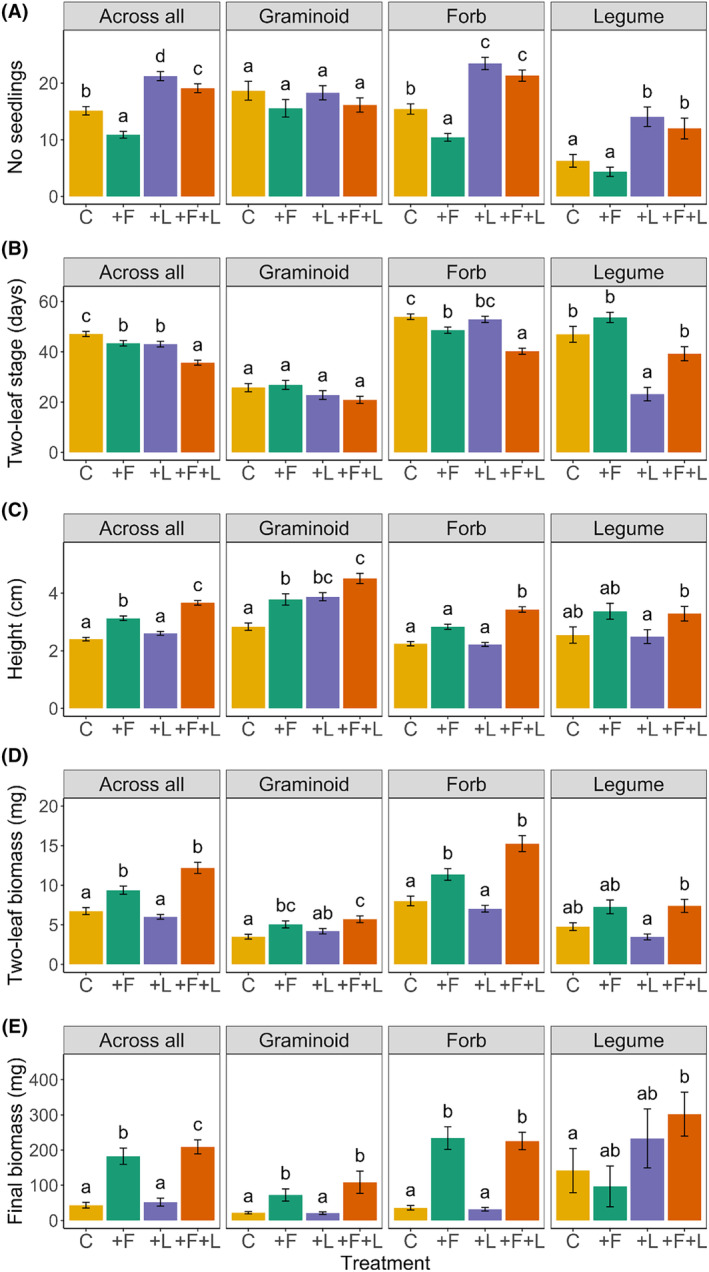
Number of seedlings (A), number of days until the two‐leaf stage was reached (measured in days from 1st March) (B), seedling height at two‐leaf stage (C), biomass at the two‐leaf stage (D), and final biomass (E) shown as means (±1 SE) per treatment (C = control, +F = fertilizer, +L = litter, and +F + L = litter and fertilizer) across all species, and separately for each functional group (graminoids, non‐legume forbs, legumes). Levels of significance are indicated by letters starting with a for the lowest mean value differentiating from other means in the same group based on the Tukey test.

The number of emerged seedlings differed among treatments. Nitrogen fertilization decreased the number of seedlings by 28% (Figure 1A), while litter cover increased the seedling number by 40%. In combination, the positive effect of litter was not outweighed by the negative response to fertilization and seedling number still increased by 26% compared to the control without litter and fertilizer addition (Table 1). The effects of litter alone and in combination with fertilization varied among functional groups (significant interaction, see Table 1). For graminoids, the treatments did not have significant effects on the number of seedlings. For forbs and legumes, litter had positive effects on the number of seedlings irrespective of additional fertilization. Fertilization alone decreased the number of seedlings in forbs, while the number of seedlings in legumes was not affected by fertilization.

**TABLE 1:** Summary of linear‐mixed effects models for the number seedlings, the proportion early‐emerging seedlings, time to reach the two‐leaf stage, seedling height, specific leaf area, two‐leaf biomass and final biomass, each variable explained by fertilization, litter, its interaction, functional group, its interaction with fertilization, its interaction with litter and its interaction with both, added sequentially.

	df	No seedlings		% early emergence		Time two‐leaf stage		Height		SLA		Two‐leaf biomass		Final biomass	
		Chi^2^	*p*‐Value	Chi^2^	*p*‐Value	Chi^2^	*p*‐Value	Chi^2^	*p*‐Value	Chi^2^	*p*‐Value	Chi^2^	*p*‐Value	Chi^2^	*p*‐Value
Fertilization	1	18.61	<0.001	16.59	<0.001	6.33	0.012	81.03	<0.001	19.10	<0.001	85.87	< 0.001	116.95	<0.001
Litter	1	51.43	<0.001	2.67	0.102	48.34	<0.001	16.06	<0.001	10.77	0.001	<0.01	0.982	<0.01	0.968
Fertilization × Litter	1	66.01	<0.001	58.09	<0.001	0.20	0.651	8.46	0.004	2.37	0.123	14.61	<0.001	16.95	<0.001
Functional group (FG)	2	4.23	0.121	14.83	<0.001	26.25	<0.001	10.75	0.005	0.74	0.690	13.27	0.001	17.84	<0.001
Fertilization × FG	2	0.16	0.921	18.55	<0.001	25.96	<0.001	9.22	0.010	5.31	0.070	7.44	0.024	25.12	<0.001
Litter × FG	2	14.32	<0.001	3.20	0.202	25.38	<0.001	14.58	0.001	4.77	0.092	3.49	0.174	7.15	0.028
Fertilization × Litter × FG	2	10.17	0.006	14.85	<0.001	16.50	<0.001	11.51	0.003	3.43	0.180	3.78	0.151	0.29	0.865

*Note*: The degrees of freedom (df) for each fixed effect is displayed. For each variable, Chi^2^ and *p*‐values are shown. For the random structure see the Section 2.

The proportion of early emerging seedlings (i.e. their change from March until May) also varied with treatment. With fertilization, a larger share of seedlings was counted in March compared to the control (Figure S3a). Irrespective of the treatment seedlings of graminoids emerged earlier than those of non‐legume forbs and legumes (Table 1), and treatment effects were only significant in non‐legume forbs.

The time until the seedling developed the first true leaves was similarly accelerated by fertilization and litter (8% and 9%, respectively). The positive effects of both factors were additive: when fertilization was combined with a litter cover, seedlings needed almost 12 days less to develop the first true leaves than seedlings in the control. Graminoids reached the two‐leaf stage faster than non‐legume forbs (Figure 1B, Table 1) and did not respond to the treatments. Within forbs, the number of days until reaching the two‐leaf stage was reduced with fertilization by approx. 5 days and this effect was even stronger in combination with litter (13.5 days). Within legumes, the litter cover halved the number of days until the two‐leaf stage was reached compared to the control, while fertilization or the combination of both did not show effects.

### 3.2 Seedling traits and biomass

On average, seedling height increased with fertilization by 30% compared to the control or the pure litter treatment. The increase in seedling height even reached 53% when fertilization and litter cover were combined. However, the strength of treatment effects varied among functional groups (Table 1). Non‐legume forbs and legumes reflected the overall results across functional groups (Figure 1C). Within graminoids, the effects of litter cover and fertilization alone were similar and increased seedling height by 33% and 37%, respectively, compared to the control. Seedlings with the combined addition of litter and fertilizer exceeded seedlings in the control in height by 59% (Figure 1C). Both, litter cover and fertilization affected specific leaf area (SLA) of the first true leaves (Table 1, Figure S3b). However, after accounting for seedling density, only fertilization remained significant (Table S2). Fertilization alone or in combination with litter increased SLA by 12% or 8%, respectively, compared to litter addition, but not to the control. Seedlings belonging to different functional groups had similar SLA in the two‐leaf stage irrespective of treatments.

At the two‐leaf stage, seedlings growing with fertilizer addition had 50% higher biomass compared to the control or pure litter addition. Biomass was even doubled when fertilizer was applied in combination with litter addition (Figure 1D). Graminoids significantly increased their biomass compared to the control with fertilizer addition and with fertilizer in combination with litter. This also holds for non‐legume forbs. In comparison however, seedling biomass of non‐legume forbs in the two‐leaf stage was significantly greater than those of graminoids in every treatment, except for the litter addition treatment. Within legumes, treatment effects were weaker, and only seedling biomass in the combined treatment of litter with fertilizer addition was higher than in the pure litter addition treatment (Figure 1D).

Over time biomass responses to fertilization became stronger (Figure 1E). Fertilization increased final biomass by 320% compared to the control. In combination with litter, fertilization led to an increase in final biomass of 400%. Further, functional groups varied in their responses (Figure 1E, Table 1). Both graminoids and non‐legume forbs responded positively to fertilization and graminoids even more in combination with litter. Final biomass increases were higher in non‐legume forbs than in graminoids. Legumes showed highly variable responses, but in general, they showed a positive response to litter addition, while the effects of fertilization even tended to be negative (Figure 1E).

### 3.3 Effect sizes

**FIGURE 2:**
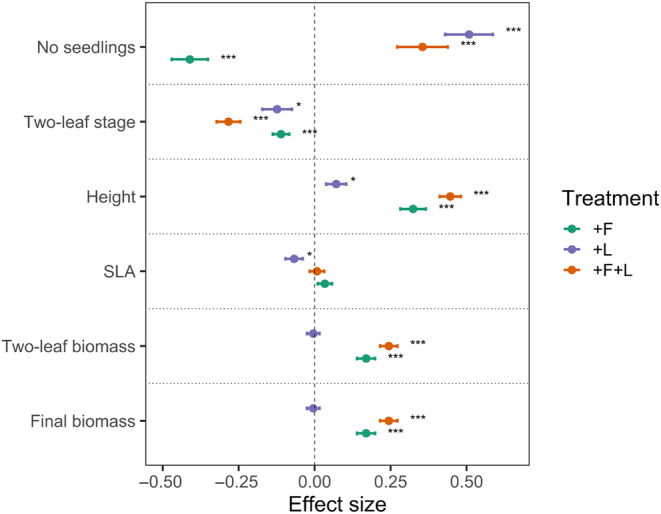
Effect sizes calculated as log response ratio (ln RR) comparing treatments (litter cover, fertilizer addition, or both) against the control (without litter or fertilizer addition) for all measured variables. Shown are means (±1 SE) across species. Positive values indicate that values measured in a treatment are larger, while negative values indicate that values measured in a treatment are smaller than in the control. Stars indicate a significant difference from the control and were calculated with the Tukey test.

Effect sizes calculated for the treatments with litter and fertilization and their combination as log‐response ratio (ln RR) against the control varied greatly among the measured variables and were dependent on the treatment. Both, fertilizer and litter showed the largest – but opposing – effects on the number of emerged seedlings (Figure 2). Treatment effects on SLA were smallest among all measured variables, and only significant for litter. For seedling height and biomass, the effect sizes of fertilizer were larger than those of litter (which were even non‐significant for seedling biomass), but in combination with a litter cover, the positive effects of fertilization on seedling height and biomass became even stronger.

### 3.4 Strength and direction of litter and fertilizer effects as related to seed mass

**FIGURE 3:**
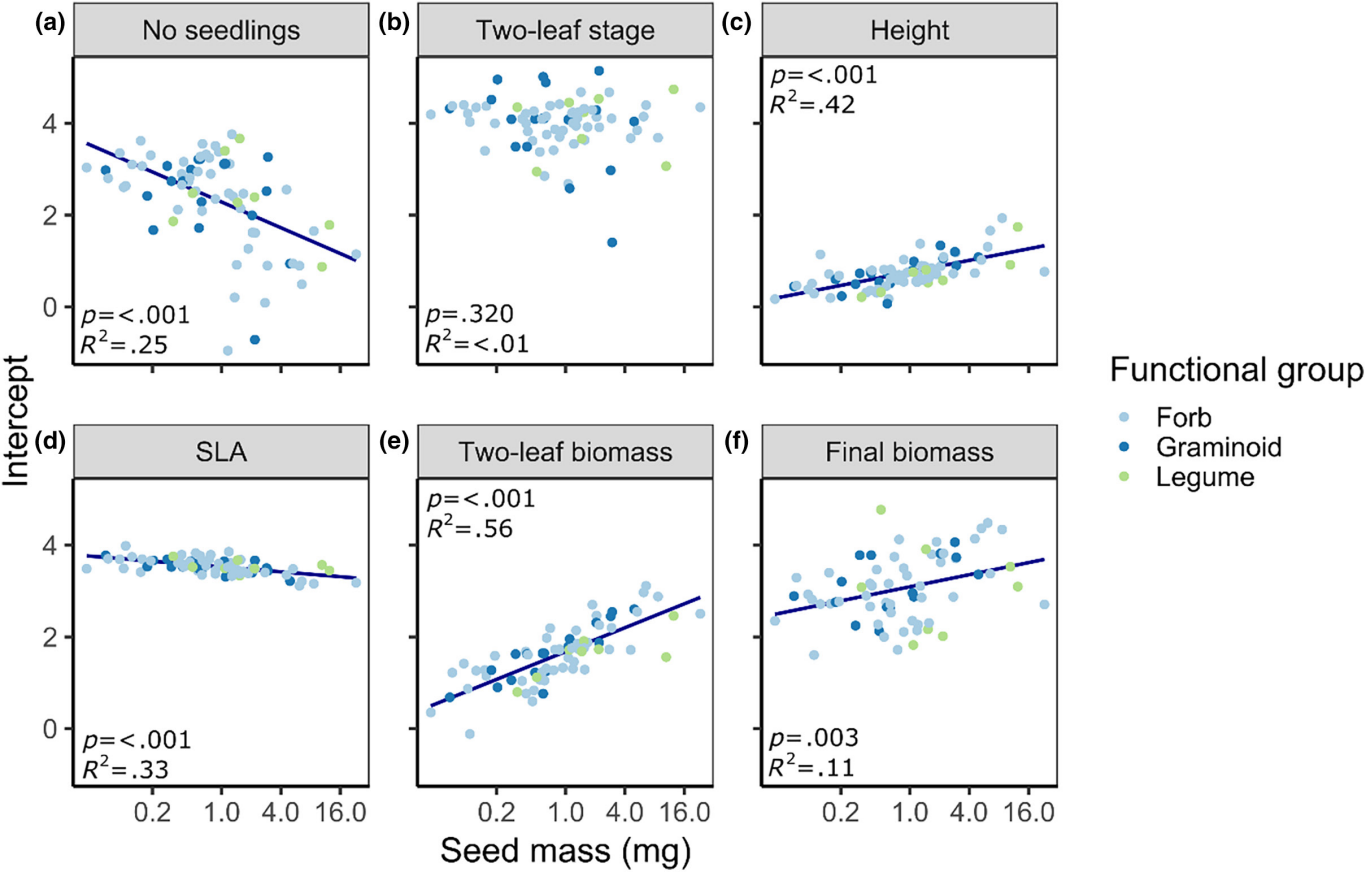
Regression models of the random intercepts of the full models (Table 1) against seed mass for (a) number of emerged seedlings, (b) days to reach the two‐leaf stage, (c) seedling height, (d) specific leaf area (SLA), (e) two‐leaf biomass, and (f) final biomass across all study species. Seed mass on the *x*‐axis is converted to log‐scale as this was done for the regressions, but axis labels still represent the real values. Regression lines were added when *p* < .05.

**FIGURE 4:**
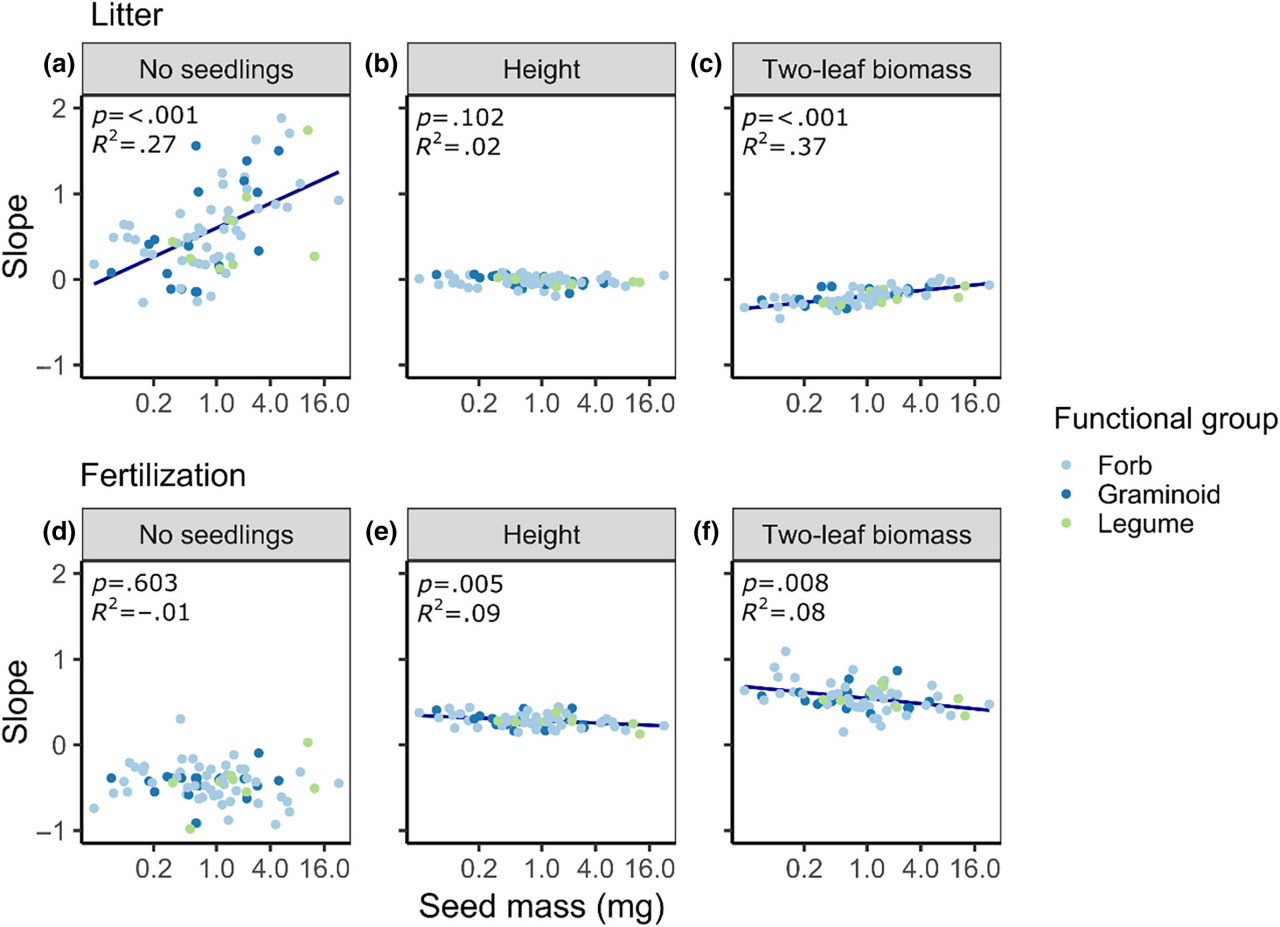
Regression models of the random slopes against litter extracted from the full models (Table 1) against seed mass for (a) number of seedlings, (b) seedling height, and (c) two‐leaf biomass, and regression models of the random slopes against fertilization extracted from the full models (Table 1) against seed mass for (d) number of seedlings, (e) seedling height, and (f) two‐leaf biomass. The Seed mass on the *x*‐axis is converted to log‐scale as this was done for the regressions, but axis labels still represent the real values. Regression lines were added when *p* < .05.

The number of emerged seedlings was lower in species with greater seed mass (Figure 3a), while the time until the seedlings developed the first true leaves varied independent of seed mass (Figure 3b). Moreover, seedlings of species with heavier seeds grew taller and had higher biomass – especially in the two‐leaf stage, while their SLA was slightly lower (Figure 3c–f). Regarding the treatments, litter cover was more beneficial for the emergence of heavier seeds, while the effects of fertilization on the number of emerged seedlings varied independent of seed mass (Figure 4a,d). Litter or fertilizer effects on seedling height were barely different among species with different seed masses, except that seedlings of smaller‐seeded species were more positively influenced in their height growth by fertilization (Figure 4b,e). Regarding seedling biomass, species with lighter seeds were rather negatively influenced by litter addition, indicated by a negative slope, while the biomass of seedlings with heavier seeds was barely affected by litter addition with a slope close to zero (Figure 4c, Figure S4a). In contrast, the seedling biomass of smaller‐seeded species was positively influenced by fertilization, while those with heavier seeds were hardly affected (Figure 4f, Figure S4b).

## 4 DISCUSSION

### 4.1 Single and combined effects of fertilization and litter

As expected from previous studies, fertilization as well as litter cover affected seedling emergence and growth. However, the results also confirm our hypothesis 1 that litter and fertilization often interact in their effects on all measured variables of seedling emergence and growth. Fertilization, which was chosen to resemble levels that are common for productive fertilized agricultural grasslands (Blüthgen et al., 2012; Olff et al., 1990), had generally negative effects on the number of emerged seedlings. Similar results have been obtained in other studies (Fenner, 2000; Kitajima & Tilman, 1996; Pérez‐Fernández et al., 2006; Sweeney et al., 2008). The reason why many studies find negative effects of nitrogen addition is not clear so far. It could be due to nitrogen toxicity, a specific inhibition, or an osmotic effect in response to the surplus of nitrogen which might prevent germination (Fenner, 2000). Another reason could be the influence of nitrogen compounds on abscisic acid and other phyto‐hormones which interact with soil microbes and influence germination and seedling development (Miransari & Smith, 2014). Another possible explanation is that osmotic effects reduce seed water absorption or lead to desiccation of the radical and subsequent death of the seedling before emergence (Hegarty, 1976). On the contrary, we found positive effects of litter cover on germination. A previous meta‐analysis by Loydi et al. (2013) showed neutral effects of litter on seedling recruitment in field studies of grassland species, while they were positive in common garden studies. The positive effects of litter on seedling emergence have often been observed under dry conditions (Loydi et al., 2013; Wellstein, 2012) concluding that positive effects of litter on seedling emergence are most likely due to its protective effects in maintaining soil moisture and preventing desiccation and evaporation, which are important factors for seed germination (Facelli & Pickett, 1991; Zhang et al., 2022). Further, litter cover could have positive effects on germination by reducing temperature extremes preventing freezing of the radicle and the cotyledons during cold nights (Facelli & Pickett, 1991; Hou et al., 2020). We could not observe any significant effect of the litter cover on mean soil temperature and its daily fluctuations (see Figure S2c). Therefore, the positive effects of litter on germination were most likely attributable to the effect on moisture and reduced evaporation. In our combined treatment adding fertilizer and litter, the negative effects of fertilization found in the separate treatment were partially mitigated through litter cover. We are not aware of further comparable studies from the literature. From our results, we argue that this mitigation of negative fertilizer effects on germination was due to a protective moisturizing effect of litter (Ruprecht et al., 2010; Wellstein, 2012) weakening the osmotic effect of nitrogen fertilization.

Regarding seedling traits and biomass, we found positive effects of nitrogen fertilization for seedling biomass and height, but not for SLA. These results are in line with studies on adult plants showing that enhanced plant growth under nitrogen fertilization is not associated with an increase in SLA (Liang et al., 2020). The positive effects of nitrogen on seedling growth indicate that seed reserves are quickly exploited and seedlings become reliant on soil nutrients for further growth.

Litter cover alone, however, did barely affect seedling traits and biomass in our study, which is in line with results reported from the meta‐analysis by Loydi et al. (2013) showing that litter does not affect seedling biomass under most experimental conditions. In contrast, litter cover increased the positive effects of fertilization on seedling biomass and height. Probably, the increased growth through fertilization was accelerated by the beneficial moisturizing effects of litter, while seedling growth with litter cover alone was nutrient‐limited. Decomposing litter has been suggested to increase nutrient availability (Facelli & Pickett, 1991). The grass litter used in our study had low nitrogen concentrations (~1%). To assess the effects of litter on nitrogen input, we used ion exchange resin bags, which we exposed on the soil surface in pots with and without litter cover. Contrary to our expectations that nitrogen could be leached out from litter, we found lower amounts of mineral nitrogen in pots with litter cover suggesting that microbial activity during decomposition as well as a reduced nitrogen deposition with litter cover led to lower nitrogen input in pots with litter cover.

### 4.2 Functional group responses to fertilization and litter

Confirming our hypothesis 2, we found differences among functional groups in their responses to litter and fertilization in all measured variables. The number of emerged seedlings in graminoids was neither affected by litter nor fertilization (Figure 1A). For both, non‐legume forbs and legumes, instead, the number of emerged seedlings was strongly influenced by litter (positive effects) or fertilization (negative effects). Contrasting with our results, a previous study in annual grasslands found that litter reduced the germination of grasses but not in non‐legume forbs or legumes (Amatangelo et al., 2008). The meta‐analysis by Xiong and Nilsson (1999), however, reported that the general negative effects of litter on seedling emergence were weaker in grasses than in forbs. Likely, the positive effects of litter on the seedling emergence of non‐legume forbs and legumes, which often have a less permeable seed coat, than grasses, can be explained by higher humidity under the litter cover increasing seed water absorption as important prerequisite for the initiation and completion of seed germination (Hull, 1973; Kigel, 2017).

Apart from the effects of litter on seedling emergence, differences among functional groups as often defined for grassland species (i.e. grasses, non‐legume forbs, legumes) are barely reported in the literature, possibly because many experimental studies focused on a small set of species. While the number of emerged seedlings (irrespective of litter and fertilization treatments) did not vary among functional groups in our study, measures of seedling growth did: graminoids needed fewer days to develop the first true leaves, their seedlings were taller but had smaller biomass in the two‐leaf stage than those of non‐legume forbs (Figure S5). Legume seedlings mostly showed intermediate characteristics.

Regarding treatment effects on the time to reach the two‐leaf stage, graminoids did not show any response, while the combination of fertilizer and litter accelerated seedling growth in non‐legume forbs, and litter alone in legumes. Possibly both, non‐legume forbs and legumes benefited from the moistening effect of litter, but the larger nutrient reserves in seed of legumes could explain their faster growth irrespective of additional nutrient supply. Graminoids, however, mostly reached the two‐leaf stage already in early spring (March), when the protective effects of litter were less important for seedling growth. Species of all functional groups had taller seedlings with a larger biomass in the two‐leaf stage in the treatments with fertilization (irrespective of litter cover), while litter alone only affected the height of graminoid seedlings without any impact on their biomass. Because graminoid seedlings reached earlier the two‐leaf stage than non‐legume forbs and legumes, their increased height could be a response to decreased light availability in the litter treatments which was less decomposed when graminoids reached the two‐leaf stage. Finally, the final biomass of seedlings (i.e. when a species reached in all treatments the two‐leaf stage) showed positive effects of fertilization in graminoids and non‐legume forbs, while there were no effects of fertilization on legume seedling biomass, possibly because a couple of nitrogen fixers might be independent of fertilization (Romanyà & Casals, 2020; Wolf et al., 2017). One caveat regarding the comparison of functional groups in our study is the higher number of species and taxonomic families of non‐legume forbs compared to grasses and legumes, however, the higher proportion of non‐legume forbs is in line with the great diversity of forbs compared to legumes and grasses in Central European grasslands.

### 4.3 Seed mass and treatment effects

As hypothesized (hypothesis 3), we found in addition to differences among functional groups that effects of litter and fertilization varied with seed mass. In line with previous studies (Jensen & Gutekunst, 2003; Ruprecht et al., 2010) larger‐seeded species benefitted more from litter in the number of emerged seedlings than small‐seeded species. One reason could be that large seeds can germinate in darkness (Jensen & Gutekunst, 2003), whereas smaller seeds could be more light‐dependent (Baskin & Baskin, 2001; Milberg et al., 2000). Moreover, the smaller amount of reserve tissue in small seeds (Westoby et al., 1996) could also explain their lower germination success under a litter cover. During seedling growth litter effects on seedling biomass were neutral for large‐seeded species but still negative for small‐seeded species. As described by Westoby et al. (1996), the seedlings of smaller‐seeded species were generally smaller in our study (Figure 3), and this “seedling size effect” could diminish their growth under a litter cover. In line with this explanation, smaller‐seeded species benefitted in their growth (i.e. seedling height and biomass, Figure 4e,f) from fertilization and showed a positive response to the added nutrients.

### 4.4 Implications

**FIGURE 5:**
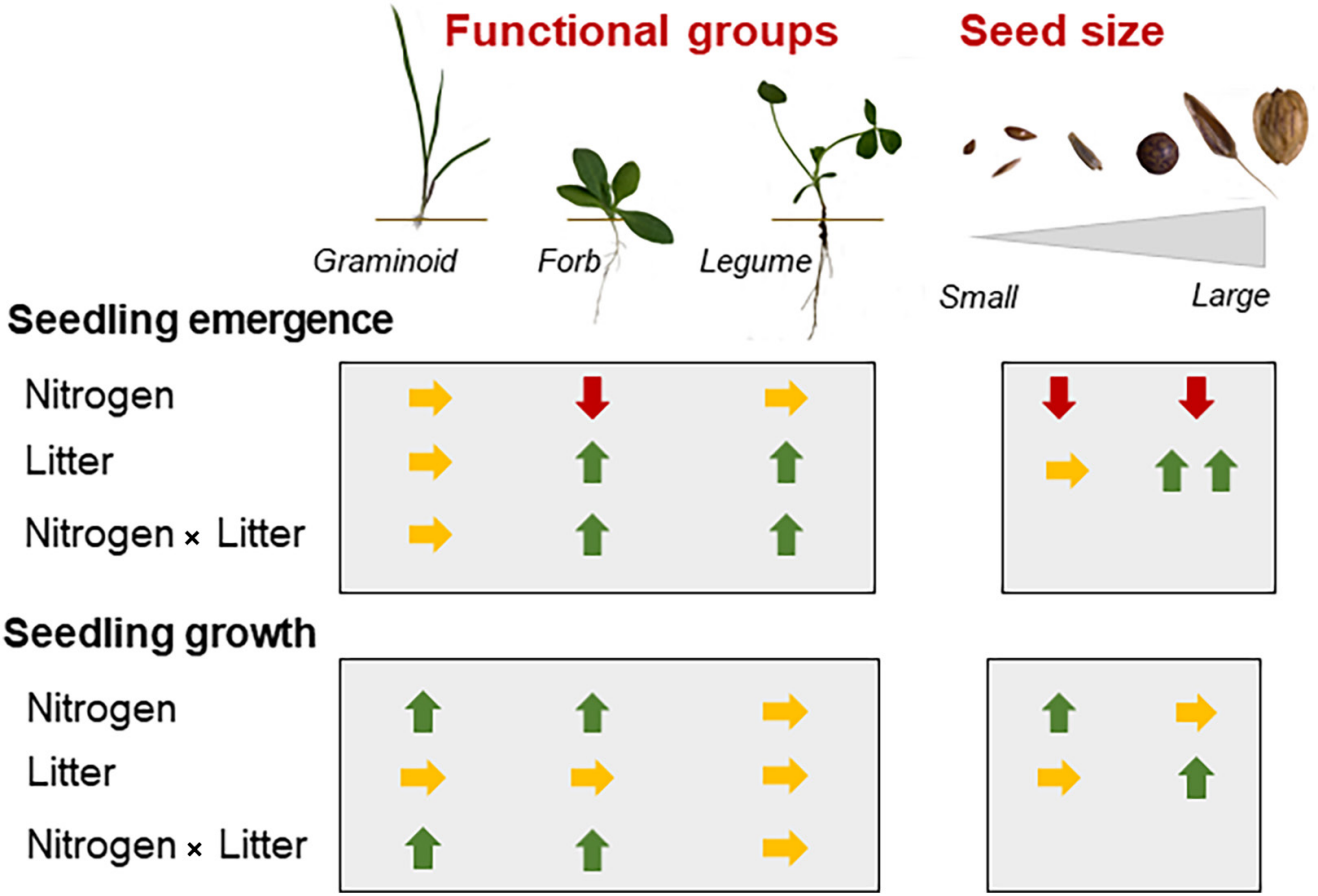
Schematic summary of the direction of litter and fertilization effects on seedling emergence and growth for each functional group (graminoids, non‐legume forbs, legumes) and depending on seed size. Vertical arrows pointing upwards (or downwards) indicate increased (or decreased) seedling emergence and growth depending on nitrogen fertilization and/or litter cover. Horizontal arrows indicate that there were no effects of nitrogen fertilization and/or litter cover.

Changes in land‐use intensity are a major cause of diversity loss in grasslands. Especially, nutrient enrichment through fertilization and anthropogenic increases in atmospheric nitrogen (Bobbink et al., 2010; Clark et al., 2007; Dickson & Foster, 2011), but also changes in management intensity – ranging from intensification to abandonment – are major drivers of diversity change (Pruchniewicz, 2017). Regeneration from seeds, i.e. seedling recruitment and growth, are key demographic processes for the persistence and stability of populations, which respond sensitively to their environment. Two potential drivers of seedling recruitment and growth, which change with land‐use intensity are nutrient availability and the amount of litter. In addition to previous studies on the single effects of litter or nitrogen, our experimental study showed that these factors interact in their effects on seedling recruitment and growth and that grassland species assigned to commonly used functional groups (graminoids, non‐legume forbs, legumes) do mostly not differ in the direction, but in the strength how they respond to these factors (as briefly summarized in Figure 5). There is some evidence from the literature that these functional groups differ in their response to changes in land‐use intensity. For example, graminoids are often reported to dominate in intensively managed grasslands (Ochoa‐Hueso & Manrique, 2010), whereas legumes react sensitively and often decline with increases in nitrogen levels (Midolo et al., 2019; Suding et al., 2005). Our results imply that these differential effects of altered land‐use intensity already play a role during the earliest stage of the plant life cycle and support calls to incorporate more plant regeneration traits in studies of community assembly (Larson & Funk, 2016). While experimental microcosms allow studying the responses of multiple species under controlled conditions, they are limited in simulating the environmental conditions in the field, where competition for light and nutrients with the established vegetation will affect the emergence and growth of the seedling. For example, it has been shown that in the short term biomass removal through mowing can partially compensate for the negative effect of fertilization on plant diversity in managed grasslands (Andraczek, Weigelt, Hinderling, et al., 2023). Based on our results, we would conclude that under high nutrient availability the effects of biomass removal would not generally be beneficial for plant diversity, but the effects could vary depending on functional groups and seed size. Thus, further studies under field conditions are required to understand the mechanisms of how changes in land‐use intensity alter biodiversity.

However, grassland restoration practices, which involve sowing on open ground or the transfer of plant material (i.e. litter), might benefit more from a better understanding of how nutrient availability and litter alone or in combination may differently affect the establishment of sown species.

## AUTHOR CONTRIBUTIONS

**Sarah Martin:** Conceptualization (equal); formal analysis (lead); investigation (lead); visualization (lead); writing – original draft (lead). **Christiane Roscher:** Conceptualization (equal); formal analysis (supporting); funding acquisition (lead); investigation (supporting); visualization (supporting); writing – original draft (supporting).

## CONFLICT OF INTEREST STATEMENT

The authors declare no conflict of interests.

## Supporting information


Appendix S1.

## Data Availability

This work is based on data elaborated by the SeedDiv Project of the Biodiversity Exploratories program (DFG Priority Program 1374). The datasets are publicly available in the Biodiversity Exploratories Information System (http://doi.org/10.17616/R32P9Q), with the DOIs: 10.25829/bexis.31627‐10; 10.25829/bexis.31678‐7; 10.25829/bexis‐31,679‐5; 10.25829/bexis.31682‐6 (data set IDs: 31627, 31678, 31679 and 31682). The R script for data analysis is available with the DOI 10.25829/bexis.31687‐10.
