# Supplementary material for: Strengths of fertilizer and litter effects on seedling recruitment and growth of grassland species differ depending on functional groups and seed size
Source: Ecol Evol. 2024 Jul 3;14(7):e11650. doi: 10.1002/ece3.11650 (PMC11220832; doi:10.1002/ece3.11650)
Supplement: Supplementary file 1 — Appendix S1. [file ECE3-14-e11650-s001.docx]

**Appendix**

Appendix 1: Methods for ion exchange resin bags

To assess input of mineral nitrogen (ammonium, nitrate) through deposition via rain during the outside period of the experiment (November to February) and via tap water used for irrigation (March to July) or leaching from litter, ion exchange resins were used. A mixture of 15 g ion exchange resins at equal proportions of strongly acidic cation exchanger (Ion Exchanger I, Merck; exchange capacity 1.4 mval mol^-1^) and strongly basic anion exchanger (Ion Exchanger III, Merck; exchange capacity 1.9 mval mol^-1^) was filled into nylon bags stabilized with a plastic ring of 5 cm diameter to get a standardized deposition area. Before exposing the bags, they were washed with distilled water and it was tested that pH was between 5 to 9. Bags were placed on the soil surface in pots without litter (four replicates) and with litter (four replicates) and pots were randomly placed in the experimental blocks. Bags were replaced after approx. seven weeks (i.e. once during the outside period of the experiment and two times during the greenhouse period). In the laboratory, the resins were eluted three times with 75 mL 2M KCl for one hour. The filtered eluate was frozen at -20°C until analysis of ammonium and nitrate.

Nitrate was analyzed photometrically after reduction to nitrite with vanadium (III) chloride in dilute hydrochloric acid. Nitrite was captured then by Griess reagents ((sulfanilamide and N-(1-naphthyl)-ethylenediamine) to produce a red dye. The absorbance was measured at 540 nm with a plate reader (Varioskan LUX, Thermo Electron LED GmbH, Osterode am Harz, Germany). An ammonium ion test kit (Spectroquant® 1.14752.0001) was used to determine the concentration of ammonium ions in a 2 M KCl sample solution. Ammonium reacts with hypochlorite under alkaline conditions to produce monochloramine, and subsequently, chloramine reacts with thymol resulting in a blue-green indophenol derivative which was determined photometrically at 655 nm.


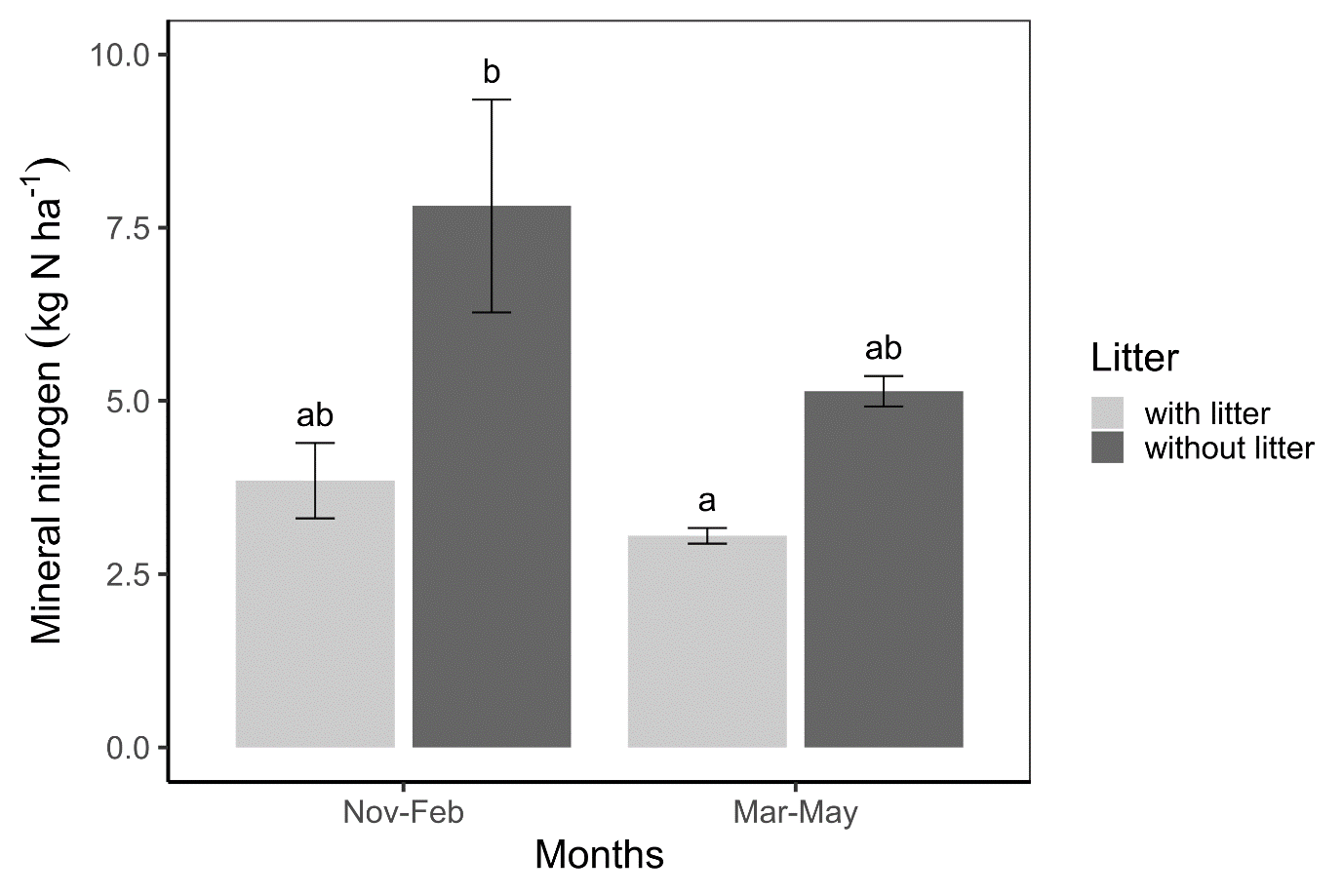


Appendix Figure S1: Mineral nitrogen extracted from ion exchange resin bags exposed on the soil surface in four pots without litter and four pots with litter. Shown is the sum of extracted ammonium and nitrate for the period from November to February (outside common garden), and March to May (open greenhouse). Differences among treatments and time periods were tested with linear mixed-effects models with litter (Chi^2^: 6.19, p= 0.013), time period (Chi^2^:2.33, p= 0.127), and their interaction (Chi^2^: 0.72, p= 0.398) as fixed effects and pot nested in block as random effects. Shown are means across the four replicates (± 1SE).


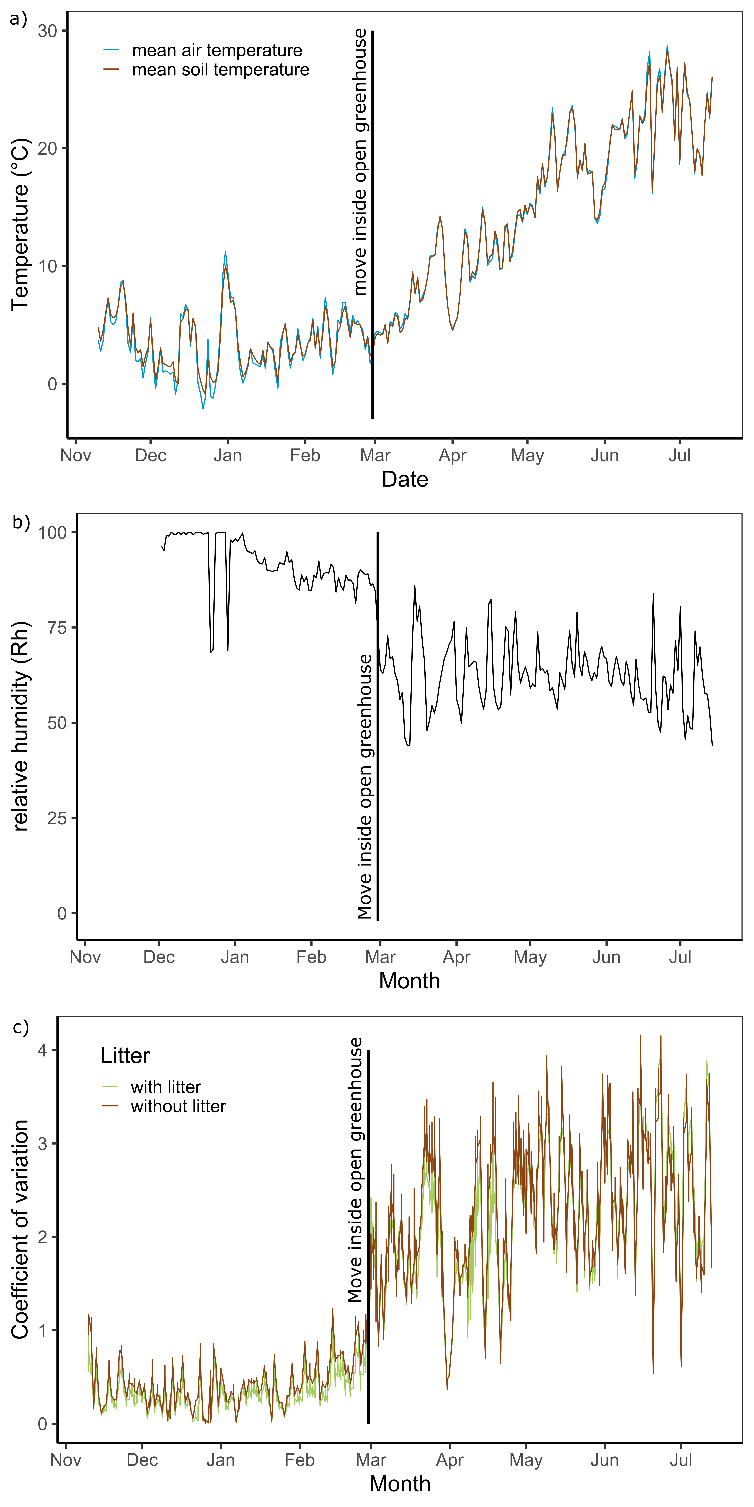


Appendix Figure S2: a) Air temperature at 4 cm above the soil surface (blue line) and soil temperature at 4 cm soil depth (brown line) measured between 10^th^ November 2021 to 14^th^ July 2022. Values are daily means of quarter-hourly measurements averaged across eight data loggers (HOBO Pro v2 Temp/6’Ext Temp, onset®) exposed in additional pots (one with litter cover and one without litter cover in each experimental block) for air and soil temperature; b) Relative humidity at 4 cm above the soil surface (black line) measured between 2^nd^ December 2021 to 14^th^ July 2022. Values are daily means of quarter-hourly measurements averaged across five data loggers (HOBO Pro v2 Temp/RH, onset®) exposed in additional pots for air temperature and relative humidity; c) Daily variation in soil temperature at 4 cm soil depth measured between 10^th^ November 2021 to 14^th^ July 2022. The coefficient of variation was calculated from the quarter-hourly measurements of the soil temperature (a) and then averaged across four data loggers (HOBO Pro v2 Temp/6’Ext Temp, onset®) exposed in additional pots one in each block. Per block there was one logger with litter cover (green line) and one without litter cover (brown line) for air and soil temperature. The solid vertical line indicates the transfer of the pot experiment from the outside common garden to the open greenhouse at the end of February.


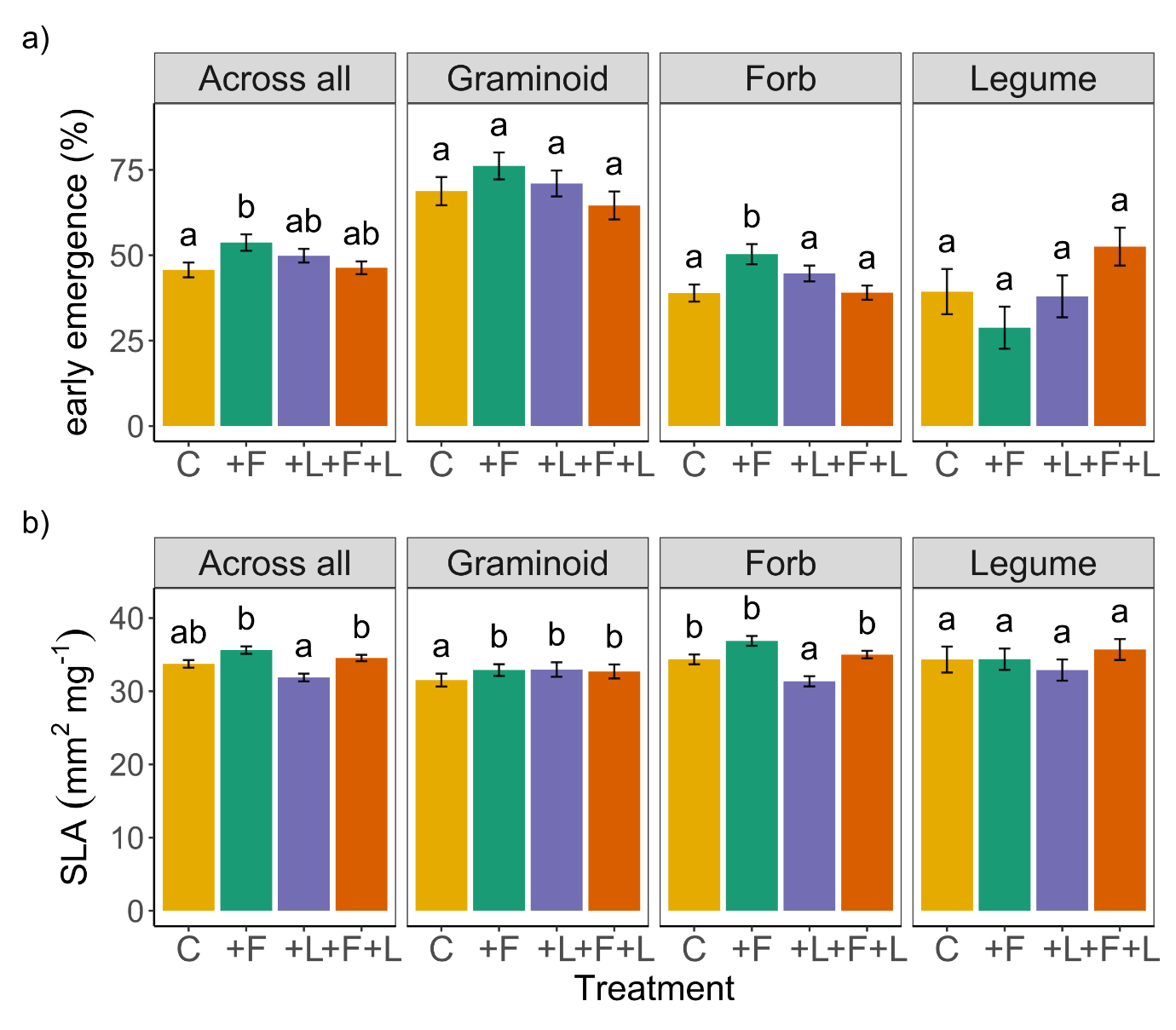


Appendix Figure S3: Proportion of early emergence (a), and specific leaf area (b) shown as means (± 1 SE) per treatment (C = control, +F = fertilizer, +L = litter, and +F+L = litter and fertilizer) across all species, and separately for each functional group (graminoids, non-legume forbs, legumes). Levels of significance are indicated by letters starting with a for the lowest mean value differentiating from other means in the same group.


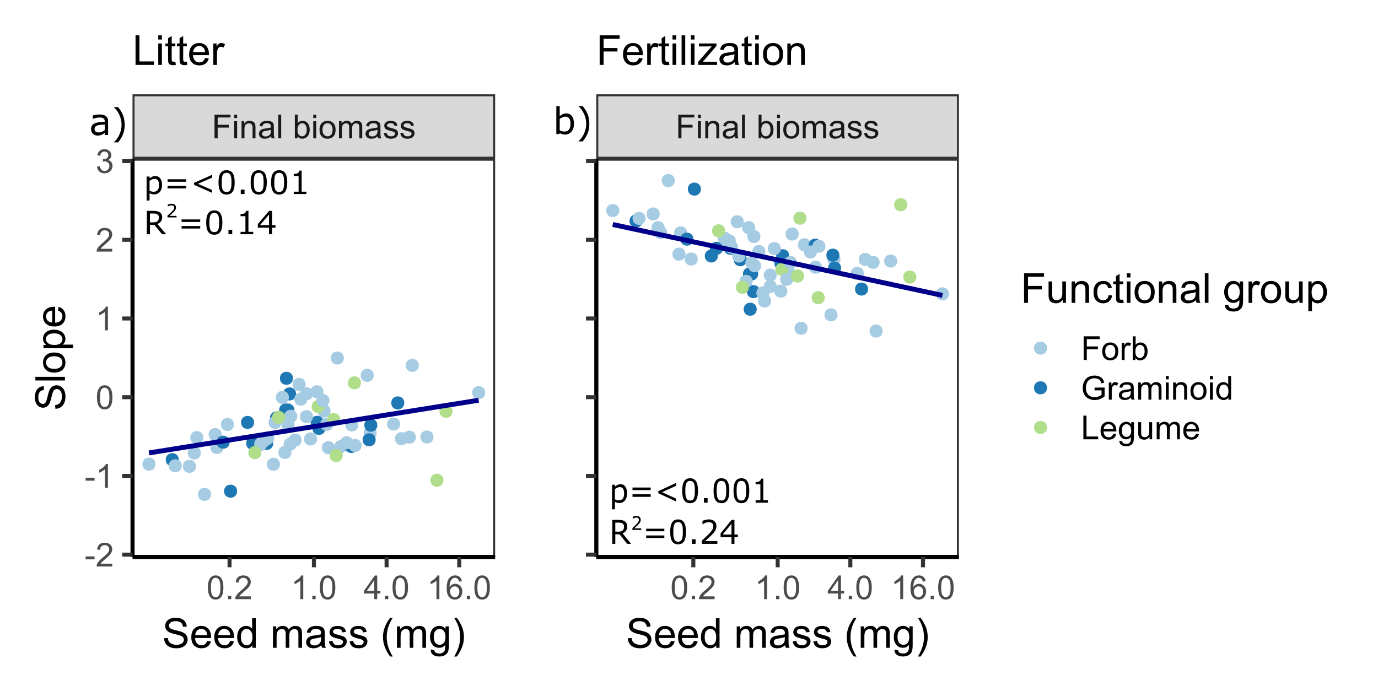


Appendix Figure S4: Regression models of the random slopes against litter extracted from the full model (Table 1) against seed mass for final biomass (a), and regression model of the random slopes against fertilization extracted from the full model (Table 1) against seed mass for final biomass (b). Seed mass on the x-axis is converted to log-scale as this was done for the regressions, but axis labels still represent the real values. Regression lines were added when p<0.05.


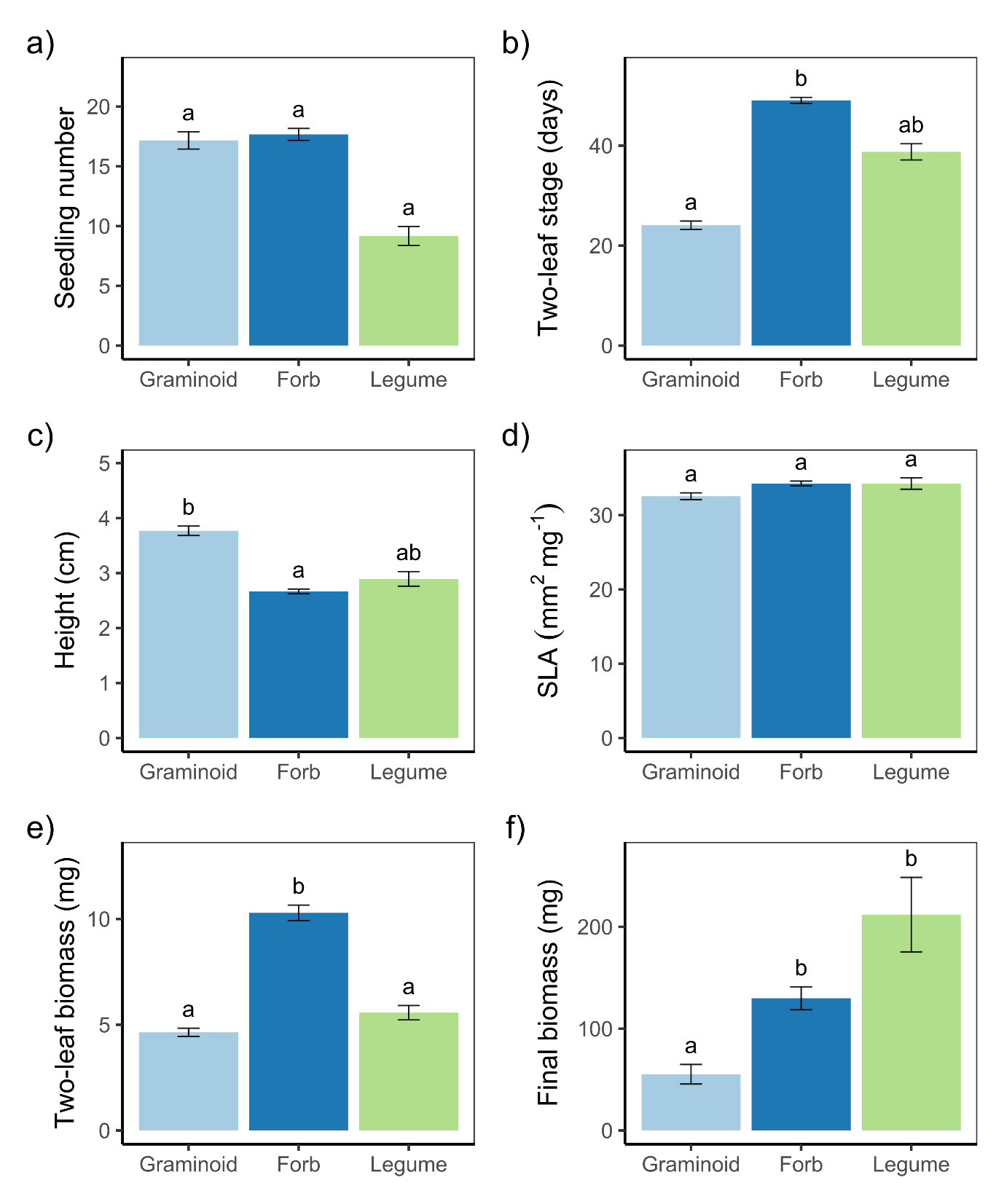


Appendix Figure S5: Seedling number (a), number of days until the two-leaf stage was reached (measured in days from 1^st^ March) (b), seedling height (c), specific leaf area (d), two-leaf biomass (e), and final biomass (f). Shown are means (± 1 SE) per functional group across all treatments. Letters indicate significant differences among functional groups based a post-hoc test calculating the pairwise contrasts of the estimated marginal mean with the *emmeans* package (Lenth, 2023).


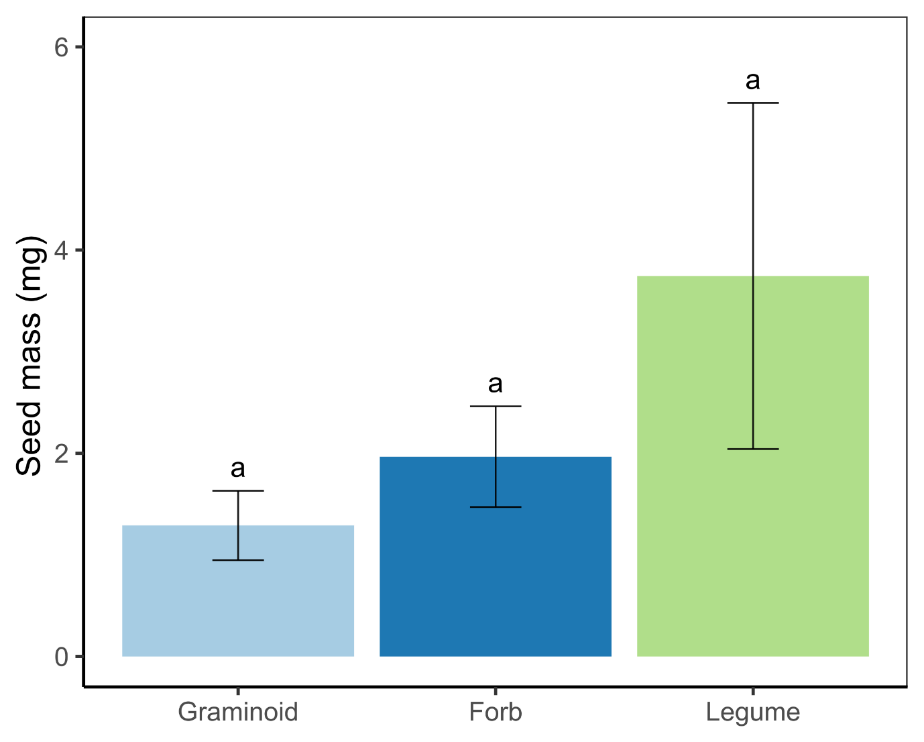


Appendix Figure S6: Seed mass per functional group (mean ± 1 SE) based on species means across regions of origin, when several seed sources were used. Letters indicate significant differences among functional groups based on the results of an ANOVA comparing the three different functional groups in their seed masses (F_2_ = 1.432, p = 0.246, df = 72).

Appendix Table S1: List of study species including family and assignment to functional groups. Additionally, the number of origins is indicated, and the mean of the seed mass (in mg) across the seeds from different origins.

| Species | Family | Functional group | No. of origins | Seed mass (mg) |
| --- | --- | --- | --- | --- |
| *Achillea millefolium* agg. L. | Asteraceae | forb | 3 | 0.152 |
| *Agrimonia eupatoria* L. | Rosaceae | forb | 3 | 23.176 |
| *Agrostis capillaris* L. | Poaceae | graminoid | 3 | 0.067 |
| *Anthoxanthum odoratum* L. | Poaceae | graminoid | 2 | 0.608 |
| *Anthriscus sylvestris* (L.) Hoffm. | Apiaceae | forb | 3 | 2.924 |
| *Arrhenatherum elatius* (L.) J. Presl et C. Presl | Poaceae | graminoid | 3 | 2.867 |
| *Betonica officinalis* L. | Lamiaceae | forb | 2 | 0.868 |
| *Briza media* L. | Poaceae | graminoid | 2 | 0.629 |
| *Bromus erectus* Huds. | Poaceae | graminoid | 3 | 4.953 |
| *Bromus hordeaceus* L. | Poaceae | graminoid | 3 | 2.961 |
| *Campanula rotundifolia* L. | Campanulaceae | forb | 3 | 0.043 |
| *Cardamine pratensis* L. | Brassicaceae | forb | 1 | 0.462 |
| *Centaurea jacea* L. | Asteraceae | forb | 3 | 1.667 |
| *Centaurea scabiosa* L. | Asteraceae | forb | 3 | 6.172 |
| *Cichorium intybus* L. | Asteraceae | forb | 3 | 1.214 |
| *Cirsium oleraceum* (L.) Scop. | Asteraceae | forb | 3 | 2.060 |
| *Clinopodium vulgare* L. | Lamiaceae | forb | 2 | 0.395 |
| *Crepis biennis* L. | Asteraceae | forb | 2 | 1.061 |
| *Cynosurus cristatus* L. | Poaceae | graminoid | 3 | 0.490 |
| *Dactylis glomerata* L. | Poaceae | graminoid | 3 | 1.065 |
| *Daucus carota* L. | Apaiceae | forb | 3 | 0.868 |
| *Deschampsia cespitosa* (L.) P. Beauv. | Poaceae | graminoid | 2 | 0.204 |
| *Dianthus carthusianorum* L. | Caryophyllaceae | forb | 1 | 0.575 |
| *Falcaria vulgaris* Bernh. | Apiaceae | forb | 1 | 1.185 |
| *Festuca pratensis* Huds. | Poaceae | graminoid | 3 | 2.045 |
| *Festuca rubra* L. | Poaceae | graminoid | 3 | 1.100 |
| *Galium mollugo* agg. Mill. | Rubiaceae | forb | 3 | 0.548 |
| *Geranium pratense* L. | Geraniaceae | forb | 2 | 6.534 |
| *Helictotrichon pubescens* (Huds.) Pilg. | Poaceae | graminoid | 2 | 2.182 |
| *Heracleum sphondylium* L. | Apiaceae | forb | 3 | 4.498 |
| *Holcus lanatus* L. | Poaceae | graminoid | 3 | 0.406 |
| *Hypericum perforatum* L. | Hypericaceae | forb | 3 | 0.107 |
| *Hypochoeris radicata* L. | Asteraceae | forb | 3 | 0.611 |
| *Knautia arvensis* (L.) J. M. Coult. | Caprifoliaceae | forb | 3 | 5.316 |
| *Lathyrus pratensis* L. | Fabaceae | legume | 2 | 10.451 |
| *Leontodon hispidus* L. | Asteraceae | forb | 2 | 1.318 |
| *Leucanthemum ircutianum* DC. | Asteraceae | forb | 3 | 0.414 |
| *Linaria vulgaris* Mill. | Plantaginaceae | forb | 3 | 0.124 |
| *Lotus corniculatus* L. | Fabaceae | legume | 3 | 1.085 |
| *Luzula campestris* (L.) DC. | Juncaceae | graminoid | 3 | 0.592 |
| *Medicago lupulina* L. | Fabaceae | legume | 3 | 1.529 |
| *Origanum vulgare* L. | Lamiaceae | forb | 3 | 0.071 |
| *Pastinaca sativa* L. | Apiaceae | forb | 3 | 2.194 |
| *Pimpinella major* (L.) Huds. | Apiaceae | forb | 2 | 1.429 |
| *Pimpinella saxifraga* L. | Apiaceae | forb | 3 | 0.653 |
| *Plantago lanceolata* L. | Plantaginaceae | forb | 3 | 1.276 |
| *Plantago media* L. | Plantaginaceae | forb | 3 | 0.362 |
| *Poa pratensis* L. | Poaceae | graminoid | 3 | 0.282 |
| *Potentilla erecta* (L.) Raeusch. | Rosaceae | forb | 1 | 0.477 |
| *Primula veris* L. | Primulaceae | forb | 2 | 1.170 |
| *Prunella vulgaris* L. | Lamiaceae | forb | 3 | 0.698 |
| *Ranunculus acris* L. | Ranunculaceae | forb | 3 | 1.563 |
| *Ranunculus bulbosus* L. | Ranunculaceae | forb | 1 | 2.766 |
| *Rumex acetosa* L. | Polygonaceae | forb | 3 | 0.754 |
| *Rumex acetosella* L. | Polygonaceae | forb | 3 | 0.397 |
| *Salvia pratensis* L. | Lamiaceae | forb | 3 | 1.868 |
| *Sanguisorba minor* Scop. | Rosaceae | forb | 3 | 4.569 |
| *Sanguisorba officinalis* L. | Rosaceae | forb | 1 | 1.540 |
| *Scabiosa columbaria* L. | Caprifoliaceae | forb | 2 | 1.360 |
| *Scorzoneroides autumnalis* (L.) | Asteraceae | forb | 2 | 0.938 |
| *Silaum silaus* (L.) Schinz et Thell. | Apiaceae | forb | 2 | 2.186 |
| *Silene flos-cuculi* L. | Caryophyllaceae | forb | 3 | 0.157 |
| *Silene latifolia* Poiret | Caryophyllaceae | forb | 3 | 0.779 |
| *Silene vulgaris* (Moench) Garcke | Caryophyllaceae | forb | 3 | 0.643 |
| *Succisa pratensis* Moench | Caprifoliaceae | forb | 1 | 1.161 |
| *Thymus pulegioides* L. | Lamiaceae | forb | 2 | 0.102 |
| *Tragopogon pratensis* L. | Asteraceae | forb | 3 | 8.638 |
| *Trifolium arvense* L. | Fabaceae | legume | 1 | 0.324 |
| *Trifolium campestre* Schreb. | Fabaceae | legume | 3 | 0.510 |
| *Trifolium medium* L. | Fabaceae | legume | 3 | 2.166 |
| *Trifolium pratense* L. | Fabaceae | legume | 3 | 1.458 |
| *Trisetum flavescens* (L.) P. Beauv. | Poaceae | graminoid | 3 | 0.177 |
| *Veronica chamaedrys* L. | Plantaginaceae | forb | 3 | 0.192 |
| *Veronica officinalis* L. | Plantaginaceae | forb | 3 | 0.093 |
| *Vicia cracca* L. | Fabaceae | legume | 3 | 12.436 |

Appendix Table S2: Summary of the mixed effects models with seedling density fitted as covariate before the experimental factors for time to reach the two-leaf stage, seedling height, specific leaf area (SLA), two-leaf biomass and final biomass. Random effects were the same as in the models without seedling density (Table 1, Methods). Each variable is explained by seedling density, fertilization, litter, its interaction, functional group, its interaction with fertilization, its interaction with litter and its interaction with both, all added one after another to each model. The degrees of freedom (df) for each fixed effect is displayed. For each variable Chi^2^ and p-values are shown.

|  |  | Two-leaf stage | | Height | | SLA | | Two-leaf biomass | | Final biomass | |
| --- | --- | --- | --- | --- | --- | --- | --- | --- | --- | --- | --- |
|  | df | Chi^2^ | p | Chi² | p | Chi² | p | Chi² | p | Chi² | p |
| Seedling density | 1 | 1.09 | 0.297 | 0.21 | 0.647 | 28.09 | <0.001 | 4.92 | 0.027 | 1.27 | 0.261 |
| Fertilization | 1 | 28.70 | <0.001 | 83.81 | <0.001 | 13.64 | <0.001 | 87.56 | < 0.001 | 117.59 | <0.001 |
| Litter | 1 | 42.74 | <0.001 | 13.49 | <0.001 | 3.46 | 0.063 | 0.70 | 0.402 | 0.22 | 0.643 |
| Fertilization x Litter | 1 | 1.09 | 0.295 | 8.16 | 0.004 | 3.76 | 0.052 | 12.73 | < 0.001 | 15.72 | <0.001 |
| Functional group (FG) | 2 | 26.16 | <0.001 | 10.67 | 0.005 | 0.32 | 0.853 | 13.79 | 0.001 | 17.41 | <0.001 |
| Fertilization x FG | 2 | 42.62 | <0.001 | 9.23 | 0.010 | 4.53 | 0.104 | 7.75 | 0.021 | 24.77 | <0.001 |
| Litter x FG | 2 | 22.96 | <0.001 | 15.15 | <0.001 | 2.27 | 0.321 | 5.33 | 0.070 | 7.22 | 0.027 |
| Fertilization x Litter x FG | 2 | 19.37 | <0.001 | 11.25 | 0.004 | 4.09 | 0.129 | 3.59 | 0.166 | 0.28 | 0.868 |
